# Molecularly imprinted polymer/CdTe quantum dots-decorated polymer optic fiber microprobes for Amoxicillin detection

**DOI:** 10.3389/fbioe.2025.1700654

**Published:** 2025-10-01

**Authors:** Jianfeng Liang, Qixuan Wu, Ran Xiao, Ruijie Yu, Hao Chen, Yangjie Tang, Jie Zhang, Chenchen Liu, Guowei Yang, Hongxiang Lei

**Affiliations:** ^1^ School of Materials Science and Engineering, Nanotechnology Research Center, Guangdong Engineering Technology Research Centre for Functional Biomaterials, State Key Laboratory of Optoelectronic Materials and Technologies, Sun Yat-sen University, Guangzhou, China; ^2^ Guangzhou Foreign Language School, Guangzhou, China

**Keywords:** polymer optic fiber microprobe, optical biosensor, molecularly imprinted polymer (MIP), quantum dots (QDs), AMX detection

## Abstract

Amoxicillin (AMX) is a widely used antibiotic for infectious diseases. However, excessive residues of AMX in the food chain and environment pose serious threats to public health, making precise monitoring of AMX crucial. Among various detection methods, fluorescence spectroscopy has garnered significant attention due to its unique advantages. Nevertheless, conventional fluorescence probes based on organic dyes or quantum dots (QDs) suffer from limitations such as difficult separation, easy pollution, poor biocompatibility and safety, lack of specificity and *in situ* detection. To address these challenges, we developed a novel sensor based on a molecularly imprinted polymer (MIP)/CdTe quantum dots-decorated polymer optical fiber microprobe (POF MP) for AMX detection. This sensor offers multiple advantages, including good specificity, reusability and stability, excellent biocompatibility and safety, *in situ* monitoring, and residue-free operation. The proposed sensor demonstrates a linear detection range of 0.5–50 μg/L with a limit of detection (LOD) of 0.31 μg/L. This innovative sensor provides a promising solution for monitoring AMX concentrations in biological and environmental systems, contributing to advancements in microenvironmental monitoring, pharmaceutical sensing, and biomedical therapeutics.

## 1 Introduction

Antibiotics are a class of widely used drugs in modern medicine, playing a crucial role in the prevention and treatment of infectious diseases (Cook and Wright, 2022). Among them, Amoxicillin (AMX) is a synthetic β-lactam antibiotic that is widely employed in treating infectious diseases in both humans and animals due to its broad-spectrum antibacterial activity, low cost, and efficacy against both Gram-positive and Gram-negative bacteria (Sun et al., 2016; Nguyen and Jang, 2022). However, with the overuse of antibiotics, bacterial resistance has continuously increased, leading to excessive residues in the food chain and environment. This, in turn, causes adverse effects such as intestinal microbiota imbalance, liver and kidney damage, and hypersensitivity reactions in humans, posing a severe threat to public health (Bacanli, 2024; Ben et al., 2019). To ensure consumer safety, the European Union has set the maximum residue limit (MRL) for AMX at 50 μg kg^-1^ in animal tissues and 4.0 μg kg^-1^ in milk. Therefore, developing efficient detection techniques for accurately monitoring AMX residues in biological fluids, food, and the environment is of paramount importance (Imran et al., 2023). Currently, various analytical methods have been developed for AMX detection, including chromatography (Riezk et al., 2023), electrochemical methods (Hrioua et al., 2021; Borhani et al., 2024), surface plasmon resonance (Yola et al., 2014; Ayankojoa et al., 2018), and spectrophotometry (Saini et al., 2015; Pawar et al., 2017; Zhang et al., 2020). Among these, fluorescence spectroscopy has garnered significant attention due to its advantages, such as rapid analysis, simple operation, low instrument cost, and minimal solvent consumption (Bacanli, 2024; Zhang et al., 2020). Although traditional organic dyes have been widely used as photoluminescent probes for detecting various target analytes via fluorescence spectroscopy (Sorouraddin et al., 2009), they suffer from drawbacks such as broad emission bands and spectral asymmetry which can affect the detection sensitivity (Resch-Genger et al., 2008). To overcome these limitations, quantum dots (QDs) have attracted significant attention in recent years due to their ideal optical properties, including size-dependent emission characteristics, narrow and symmetric emission peaks, long fluorescence lifetime, excellent photochemical stability, and good water dispersibility (Xue et al., 2020; Wei et al., 2016). These features make QDs commonly used as photoluminescent probes for the detection of ions, molecules, proteins, and cells (Bunkoed and Kanatharana, 2015; Nurerk et al., 2016). However, antibiotics with compounds of the same class often exhibit similar chemical structures. For instance, AMX and ampicillin, both belonging to the β-lactam antibiotic class, share a similar chemical structure. Consequently, analogous reactions with QDs will be produced, making it difficult to distinguish between them. To achieve specific detection, QDs must be modified with targeted specificity materials. Among them, molecularly imprinted polymer (MIP) represents an effective strategy for the surface modification of QDs (Wei et al., 2016). MIP is an affinity polymer synthesized for specific target molecules, offering advantages such as simple preparation, low cost, and high stability (Piletsky et al., 2012). It is generally prepared by polymerizing functional monomers and cross-linking monomers in the presence of template molecules (target analytes), resulting in a highly cross-linked three-dimensional polymer network (Piletsky et al., 2012; Turiel and Martín-Esteban, 2010). After the polymerization, the template molecules are removed, creating specific recognition cavities (Chao et al., 2016). Currently, MIP-functionalized QDs have been developed as photoluminescent probes for the selective detection of histamine (Kim and Chang, 2016), malachite green (Wu et al., 2017), chlorpyrifos (Ren et al., 2015), alpha-fetoprotein (Tan et al., 2015), and ciprofloxacin (Liu et al., 2022), which imply its feasibility to detect AMX. However, the existing MIP-based detection methods typically involve mixing the MIP with the sample solution of target analytes, making it difficult to recover the sample solution and leading to residual contamination and hindering *in-situ* detection. Additionally, these methods require large sample volumes for analysis; moreover, most QDs have certain biological toxicity and its direct contact with the target analytes or its residue in the surrounding environment can cause certain biological damage. Therefore, it is highly demanded to develop an efficient detection method of AMX with high sensitivity, good specificity, reusability and stability, excellent biocompatibility and safety, *in situ* monitoring, pollution-free and residue-free operation.

In this work, we design and develop a MIP/CdTe QDs-decorated polymer optical fiber microprobe (POF MP) to achieve a sensitive, biosafe, specific, residue-free and *in-situ* detection of AMX. Taking AMX as the template molecules, MIP-coated CdTe quantum dots (MIP-QDs) are firstly synthesized as photoluminescent material by using sol-gel copolymerization method. The prepared MIP-QDs, as functional materials, were subsequently decorated onto the surface of the POF MP via free radical photopolymerization. Thus, a novel AMX sensor is successfully fabricated and it exhibits excellent bio-safety, sensitivity, reversibility, reusability, specificity, and structure stability in AMX detection, providing a new strategy for biological microenvironment monitoring, pharmaceutical sensing, and biomedical applications.

## 2 Results and discussion

### 2.1 Design principle of MIP/CdTe QDs-decorated POF MP

**FIGURE 1:**
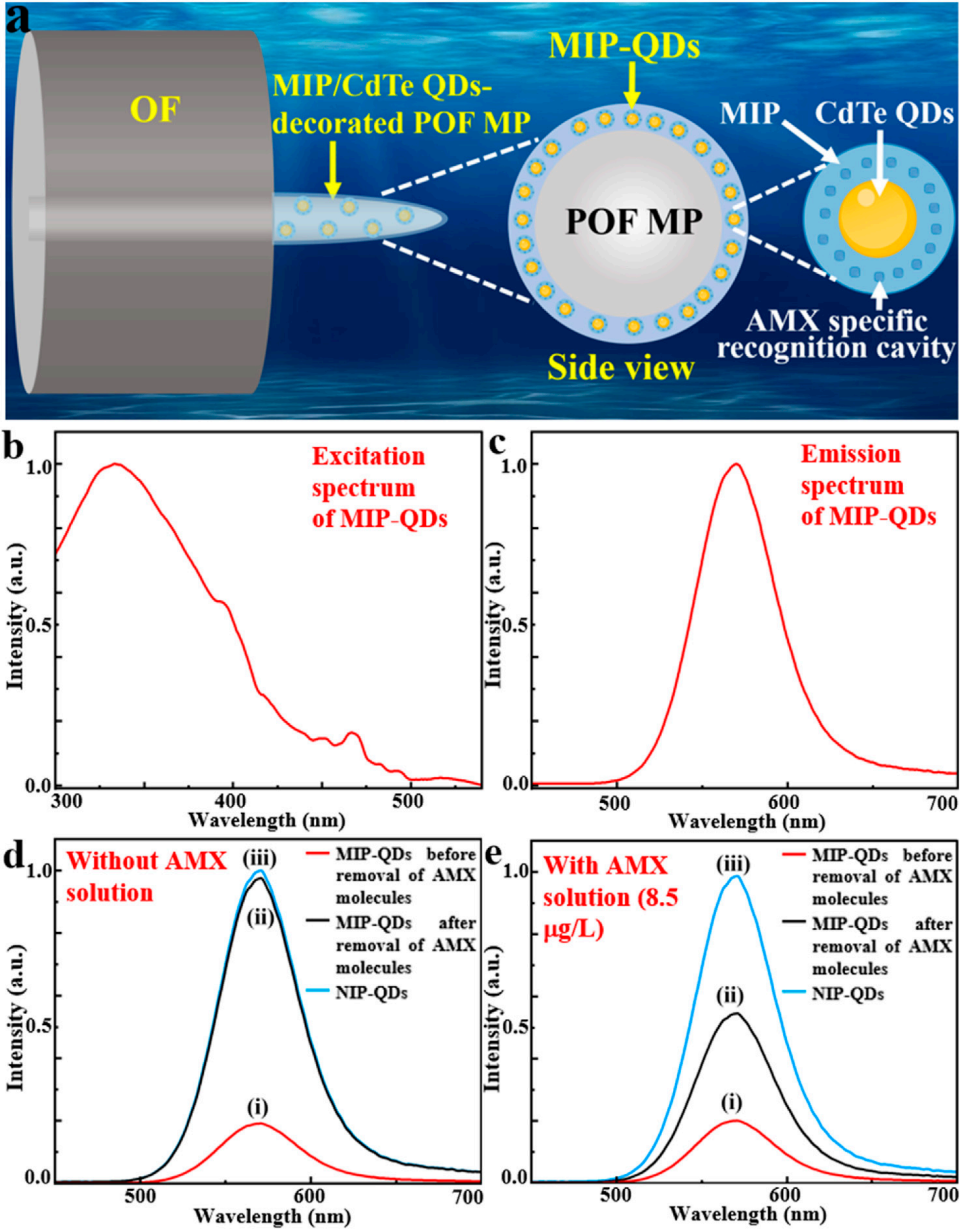
Design principle of MIP/CdTe QDs-decorated POF MP. **(a)** Schematic diagram of designed structure. **(b,c)** Excitation **(b)** and emission **(c)** spectra of MIP-QDs. **(d,e)** PL spectra in the absence **(d)** and the presence **(e)** of AMX solution (8.5 μg/L) for MIP-QDs before (i) and after (ii) the removal of AMX template molecules and for NIP-QDs (iii).

The designed MIP/CdTe QDs-decorated POF MP is schematically shown in Figure 1a, which presents a core-shell structure. The core layer is a conical POF MP fabricated at the end of a standard single-mode optical fiber (OF) using photopolymerization method; it had excellent optical transparency and bio-safety and can be served as the flexible skeleton of the AMX sensor. As the main component of the shell layer, MIP-coated CdTe quantum dots (MIP-QDs) synthesized via a sol-gel copolymerization method plays a crucial role in the AMX detection, which is decorated onto the surface of the POF MP via the photopolymerization method; it integrates the MIP with AMX specific recognition cavities and CdTe QDs with excellent luminescent properties and can be served as the flexible sensing layer for the AMX detection. The above design of core-shell structure offers high flexibility, compactness and structural stability, excellent biosafety and specificity, which is conducive to a safe, pollution-free, residue-free, specific, precise and *in-situ* detection for AMX. In principle, when a laser beam with a 375 nm wavelength (see the excitation spectrum of MIP-QDs in Figure 1b) is injected into the OF and then transmitted to the POF MP end, the MIP-QDs inside the shell layer will be irradiated and then emit a 570-nm-wavelength fluorescent light (see the emission spectrum of MIP-QDs Figure 1c). The fluorescence intensity is very sensitive to the concentration of AMX solution because of the fluorescence quenching effect. Specifically, as effective electron acceptors, AMX molecules prevent the recombination of free electrons in the conduction band with holes in the valence band in CdTe QDs. To demonstrate the high sensitivity of fluorescence intensity to AMX concentration, three samples, including MIP-QDs before and after removal of AMX template molecules and non-imprinted polymer-coated CdTe QDs (NIP-QDs), were synthesized (see Method for the detailed synthetic process); their photoluminescence (PL) spectra without and with AMX solution (8.5 μg/L as an example) were measured, as shown in Figures 1d,e. The results show that, the PL intensity of MIP-QDs before the removal of AMX molecules is the lowest in the absence of AMX solution (Figure 1di), which is attributed to the fluorescence quenching effect of AMX molecules on CdTe QDs; it is increased by 5.1% in the 8.5 μg/L of AMX solution (Figure 1ei), which is mainly because a small portion of AMX template molecules inside the MIP dissolved into the solution and the number of binding sites available for AMX molecules reduced. By contrast, the PL intensity of MIP-QDs after the removal of AMX molecules is significantly increased because of the absence of AMX molecules (Figure 1dii), which approaches that of NIP-QDs (Figure 1diii); it also indicates that the AMX template molecules were almost completely removed from the recognition cavities of MIP-QDs. However, it is decreased by 41.6% in the 8.5 μg/L of AMX solution (Figure 1eii), demonstrating the specific recognition capability of MIP-QDs toward AMX template molecules. For the NIP-QDs in the 8.5 μg/L of AMX solution (Figure 1eiii), its PL intensity remains basically unchanged and consistent with that in Figure 1diii. From the above, it is concluded that the proposed MIP-QDs with AMX specific recognition cavities exhibit the high sensitivity to AMX concentration. Therefore, through the aforementioned design, the MIP/CdTe QDs-decorated POF MP possessing excellent compactness, flexibility, bio-safety can be used to achieve a sensitive, specific, and *in situ* detection of AMX.

### 2.2 Fabrication and characterization of MIP/CdTe QDs-decorated POF MP

**FIGURE 2:**
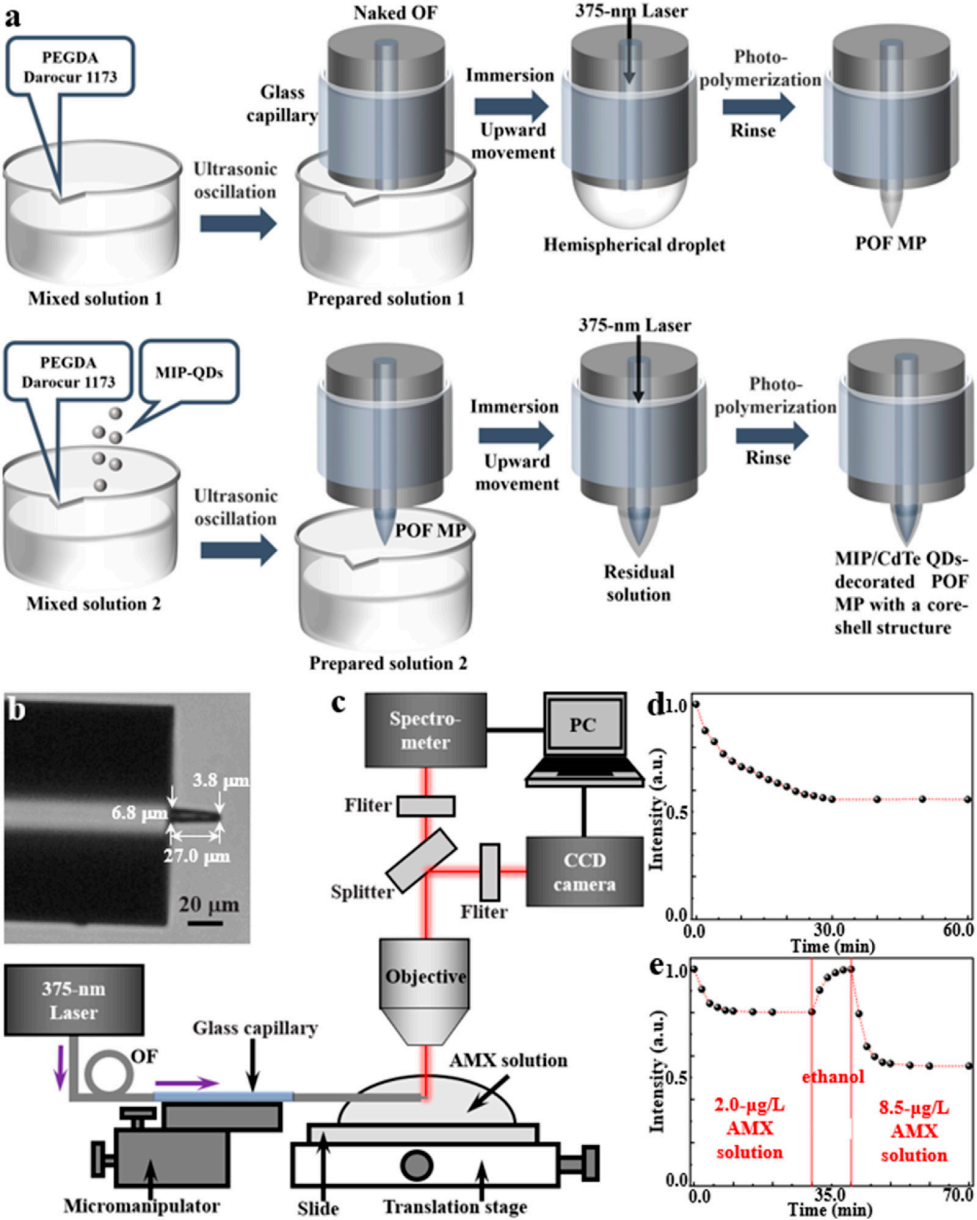
Fabrication and characterization of MIP/CdTe QDs-decorated POF MP. **(a)** Schematic of the fabrication process. **(b)** Optical microscopic image (tip diameter: 3.8 μm, bottom diameter: 6.8 μm, length: 27.0 μm). **(c)** Schematic of experimental setup for AMX sensing. **(d,e)** Response time measurements for the none-core-shell structure **(d)** and core-shell structure **(e)** sensor.

The fabrication process of MIP/CdTe QDs-decorated POF MP is schematically shown in Figure 2a and it mainly includes the fabrication of POF MP and the decoration of MIP-QDs on the surface of the POF MP, which is described below.

The POF MP was fabricated at the at the end of a standard single-mode OF via a free radical photopolymerization reaction. It can typically transform a liquid monomer solution into a solid polymer by a chain reaction process triggered by the free radicals generated from the light absorption of photo-initiator (Wu et al., 2018; Liang et al., 2025). In the experiment, polyethylene glycol diacrylate (PEGDA) was selected as the multifunctional acrylate monomer, while 2-hydroxy-2-methylpropiophenone (Darocur 1,173) was used as the photo-initiator. Due to the high absorption of Darocur 1,173 within the ultraviolet spectral range, a 375-nm-wavelength laser beam (the same wavelength with the excitation wavelength of the proposed MIP-QDs) was used to irradiate the photo-initiator. Once absorbing light energy, the photo-initiator will undergo homolytic cleavage, producing benzoyl radicals and methyl radicals. These radicals act as active centers, initiating the ring-opening polymerization of the acrylate double bonds in PEGDA. Firstly, the mixed solution one of PEGDA and Darocur 1,173 at a mass fraction of 95%:5% was ultrasonically oscillated into preparation solution 1 with a suitable viscosity. Secondly, a naked OF (core diameter: 9 μm, cladding diameter: 125 µm) with a flat end, which was processed using a fiber stripper and an OF cleaver, was placed straight down and immersed in the prepared solution one using a OF micromanipulator; before its processing, the naked fiber end needed to be sheathed using a glass capillary (inner diameter: 0.5 mm) to prevent its bending or breaking; the other end of the OF was connected to a laser source (wavelength: 375 nm). Thirdly, by moving the micromanipulator upward, a hemispherical droplet of the solution formed on the flat end of the naked OF; turning on the laser source with a 1 μW optical power, the droplet would be irradiated, inducing its photopolymerization effects and solidifying the areas in the droplet irradiated by the light into a microprobe. The polymerization reaction lasts for several seconds to several tens of seconds, with longer durations yielding thicker probes (Liang et al., 2025). After the polymerization, the residual unpolymerized solution was thoroughly rinsed multiple times with ethanol. Thus, a POF MP was formed at the end of the single-mode OF.

Subsequently, the prepared MIP-QDs (see Methods for detailed synthetic process) were decorated on the outside surface of the POF MP to produce a core-shell structure by reusing the photopolymerization method. The mixed solution two was firstly prepared by adding the MIP-QDs into the mixed solution 1 with a mass ratio of 1:3000 and then it was ultrasonically oscillated into preparation solution 2 with a suitable viscosity. Then, the POF MP was immersed in the preparation solution 2. By moving the micromanipulator upward, a residual layer of the solution adhered to the outside surface of POF MP. Similarly, the 375 nm laser beam was transmitted through the OF to irradiate the residual solution and induce its photopolymerization. After 15 s, the unpolymerized residue was rinsed with ethanol multiple times and a MIP/CdTe QDs-decorated POF MP with a core-shell structure was successfully fabricated at the end of the single-mode OF, as shown in the optical microscopic image in Figure 2b. Here, the tip diameter, the bottom diameter and the length of the MP are 3.8, 6.8 and 27.0 μm, respectively. As a comparison, another MIP/CdTe QDs-doped POF MP (non-core-shell structure) was also fabricated by doping MIP-QDs inside the POF MP; its fabrication process is similar to the above prepared procedure of POF MP, except that the mixed solution two was instead of solution 1.

To evaluate the AMX sensing performance of the prepared MIP/CdTe QDs-decorated POF MP, a testing system was established based on a microspectrophotometer (20/20 PV, CRAIC, Germany), as shown in Figure 2c. This system was primarily consisted of an optical microscope, a charge-coupled device (CCD) camera, and a spectrometer. The MIP/CdTe QDs-decorated POF MP end of the OF was fixed onto the translation stage of the microspectrophotometer using a micromanipulator and immersed in AMX solutions of different concentrations. A 375-nm laser was coupled into the other end of the OF to excite the MIP-QDs decorated onto/into the POF MP. The fluorescence emitted by the MIP-QDs was collected by the microscope objective and splitted into two light paths. One was directed to a color CCD camera for real-time observation and image acquisition, and the other was directed to a spectrometer to obtain PL spectra; then both of them were displayed on a computer (PC).

Based on the above experimental platform, the response time of the prepared MIP/CdTe QDs-decorated POF MP above was firstly investigated by measuring its fluorescence intensity change in AMX solution. This is an essential step before the sensor is officially put into use, which can ensure the complete binding between the AMX molecules and the recognition sites of MIP-QDs in AMX sensing and the accuracy of the AMX sensor. As a comparison, Figure 2d shows the normalized PL intensity change of the MIP/CdTe QDs-doped POF MP sensor with the non-core-shell structure starting from the moment (*t* = 0) of its contact with AMX solution (taking 8.5 μg/L concentration as an example). Obviously, the PL intensity gradually decreased until it reached a steady state at *t* = 30 min. The long response time was mainly caused by the slow diffusion of AMX molecules into the whole recognition sites of MIP-QDs doped inside the sensor. By contrast, the proposed MIP/CdTe QDs-decorated POF MP sensor with the core-shell structure displayed a faster response time, as shown in Figure 2e (taking 2.0 μg/L AMX solution as an example). The decrease in PL intensity could be observed within just 2 min; approximately 80% of the total signal response occurred within 4 min and it reached an equilibrium (>98%) in approximately 10 min. The obvious decrease in the response time was mainly attributed to the much shorter diffusion distance required for AMX molecules in this sensing structure. Beside this faster response, the excellent reversibility and the response time stability of the sensor structure were also demonstrated, as shown in Figure 2e. Once the 2.0 μg/L AMX solution was replaced by alcohol at *t* = 10 min, the PL intensity would increase rapidly due to the removement of the AMX template molecules; about 10 min later, the PL intensity returned to its original level (*t* = 0). At this time, the alcohol was removed and another different concentration of AMX solution was added into the sample cell (taking 8.5 μg/L AMX solution as an example); it shows a similar change trend of PL intensity and a stable response time (10 min). The above further confirms that the proposed core-shell structure and detecting method enables fast, stable and reversible measurement of analytes. In the following sensing experiments, the AMX sensor used specifically refers to the MIP/CdTe QDs-decorated POF MP with the core-shell structure.

### 2.3 AMX sensing application of MIP/CdTe QDs-decorated POF MP

**FIGURE 3:**
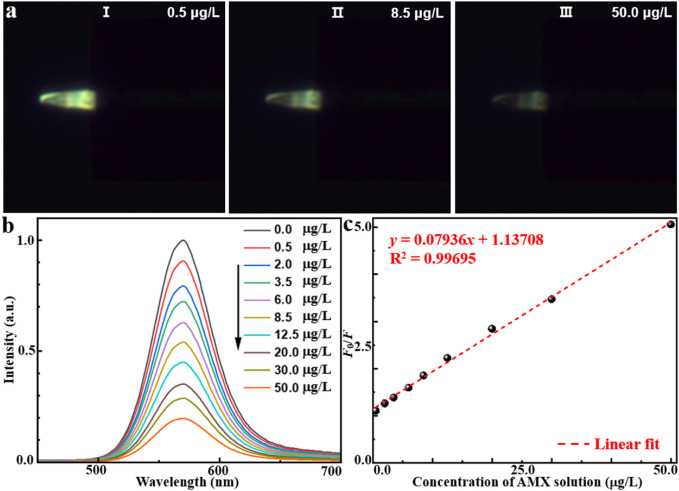
AMX sensing application of MIP/CdTe QDs-decorated POF MP. **(a)** Three representative dark-field microscope images in the AMX solution with the concentration of 0.5 (I), 8.5 (II), 50 μg/L (III). **(b,c)** PL spectra of the MIP/CdTe QDs-decorated POF MP **(b)** and the fluorescence intensity at the peak **(c)** at different concentrations of AMX solution with the range from 0.5 to 50 μg/L.

To experimentally validate the effectiveness of AMX sensing, the proposed MIP/CdTe QDs-decorated POF MP were immersed in standard test solutions with different AMX concentrations at room temperature (25 °C) to observe fluorescence intensity variations. As examples, Figure 3a (I–III) presents three representative dark-field microscopic images corresponding to AMX concentrations of 0.5, 8.5, and 50 μg/L, respectively. Obviously, the fluorescence intensity decreased as the AMX concentration increased. To further investigate the quantitative relationship, more AMX sensing experiments were performed and the corresponding PL spectra were collected with the AMX concentration range from 0.5 to 50 μg/L, as shown in Figure 3b. When the sensor was excited with a 375 nm laser beam, the emission peak could be observed at 570 nm under the different AMX concentrations, which is consistent with that of MIP-QDs in Figure 1c. Furthermore, the sensor exhibited a high sensitivity to AMX concentration; the fluorescence intensity significantly decreased with the increasing AMX concentration, which is attributed to the fluorescence quenching effect mentioned above. Figure 3c presents the quantitative relationship between the fluorescence intensity ratio (*F*_0_/*F*) and various AMX concentration. Here, *F*_0_ and *F* refer to the intensity of emission peak under zero and different AMX concentrations, respectively. A strong linear correlation was obtained, with a fitted equation of *y* = 0.07936*x* + 1.13708 and a regression coefficient of *R* ^2^ = 0.99695. Based on the 3σ criterion (Cheng et al., 2018) (σ represents the standard deviation of blank measurements and it was measured to be 0.00823 in this study), the theoretical limit of detection (LOD) could be calculated to be about 0.31 μg/L, which is comparable or superior to those obtained by other AMX detection methods (Riezk et al., 2023; Borhani et al., 2024; Zhang et al., 2020; Wong et al., 2020; Lopez et al., 2021; Chullasat et al., 2018). In conclusion, the proposed MIP/CdTe QDs-decorated POF MP can be applied for the quantitative measurement of AMX concentration with wide linear detection range and a low LOD.

**FIGURE 4:**
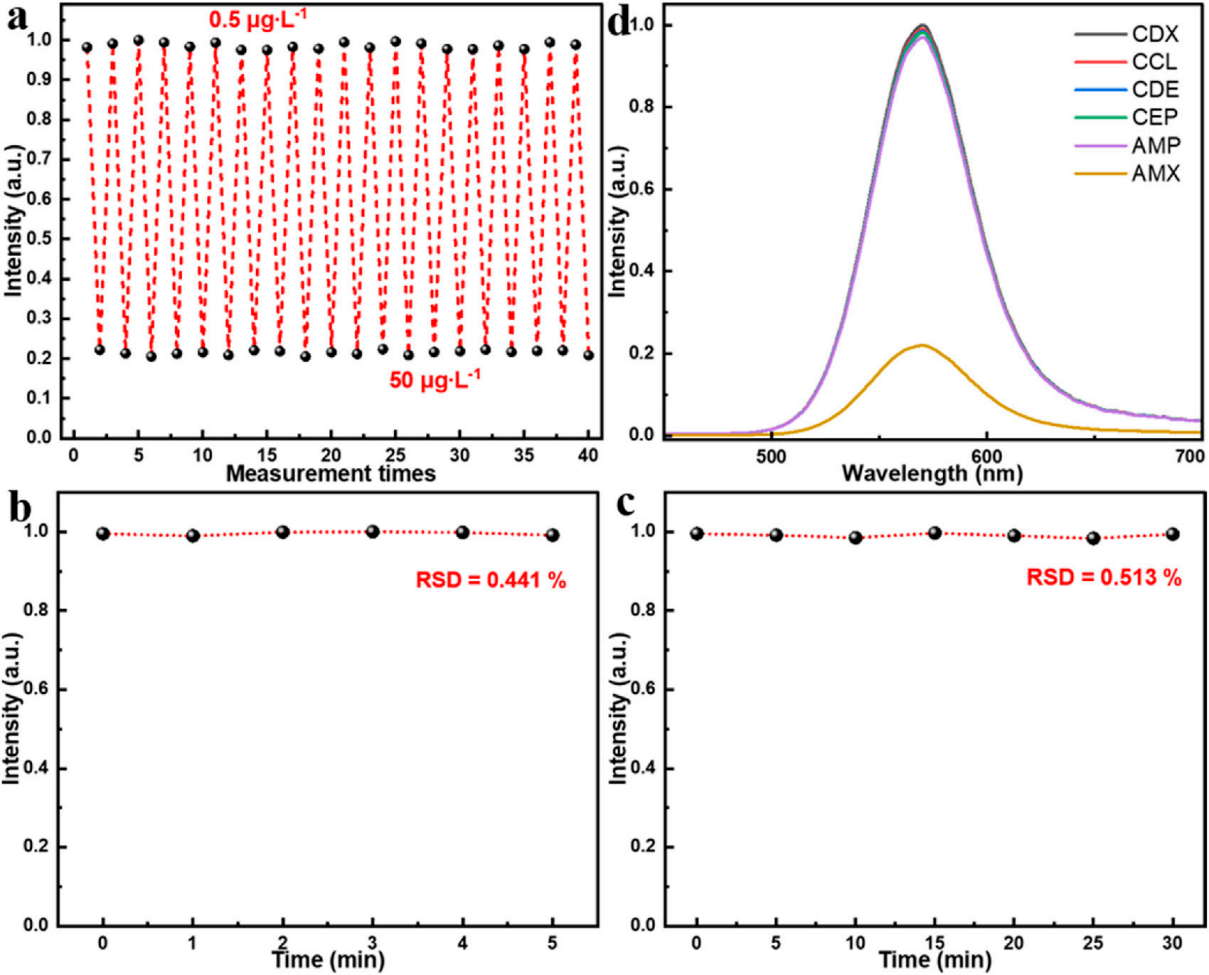
Performance test of MIP/CdTe QDs-decorated POF MP sensor. **(a)** Reusability and reversibility tests. **(b,c)** Stability tests in 5 min **(b)** and 30 min **(c)**. **(d)** Specificity tests in the same concentration (50 μg/L) of AMX solution and other antibiotics solutions with similar molecular structures.

To further evaluate the sensing performance of the proposed MIP/CdTe QDs-decorated POF MP, its reusability and reversibility, stability, and specificity have been also investigated, as shown in Figure 4. By alternately immersing the prepared MIP/CdTe QDs-decorated POF MP in AMX solutions with concentrations of 0.5 μg/L and 50 μg/L, the reusability and reversibility were firstly tested. Figure 4a presents the measured fluorescence intensity at the peak wavelength over 20 consecutive cycles. The sensor exhibited a highly responsive reaction to AMX concentration changes and no significant fluctuations in fluorescence intensity after each detection cycle was observed. The relative standard deviation (RSD) of fluorescence intensity was calculated as 1.37% at 0.5 μg/L and 1.12% at 50 μg/L, indicating that the proposed AMX sensor possesses high reversibility and reusability. Additionally, to assess the time stability of the proposed AMX sensor, multiple repeated experiments were conducted in the same concentration of AMX solution using the same MIP/CdTe QDs-decorated POF MP. Taking the AMX solution with 8.5 μg/L concentration as an example, Figures 4b,c show the six repeated measurements conducted at 1-min intervals within 5 min and the seven repeated measurements over a period of 30 min with a time interval of 5 min, respectively. The RSD of fluorescence intensity was calculated as 0.441% over 5 min and 0.513% over 30 min, demonstrating the excellent stability of the proposed AMX sensor. Finally, the specificity of the sensor was also investigated by comparing their PL spectra after the interaction with the same concentration (50 μg/L as an example) of target analyte (AMX) and other antibiotics, as shown in Figure 4d. The potential interfering antibiotics used in this study included ampicillin (AMP), cephalexin (CEP), cefaclor (CCL), cefradine (CDE), and cefadroxil (CDX); all of them belong to the β-lactam antibiotic class, sharing the same or similar side-chain structures with AMX, and they are capable of undergoing similar interactions with CdTe QDs. From Figure 4d, it can be seen that, the sensor exhibited a strong response to AMX, whereas its response for other antibiotics were significantly weaker, which demonstrates the excellent specificity of the sensor for AMX detection. It was mainly attributed to numerous specific recognition sites of AMX molecule on the surface of CdTe QDs, leading to a pronounced fluorescence quenching. This also inspires us that, selecting specific template molecules and appropriate functional materials during the preparation process of the sensor structure can further expand the detection range of target analytes (such as drugs, hormones, toxins, proteins, etc.), which will promote the development of biomedicine and environmental monitoring.

### 2.4 Discussion

Compared with other reported methods for AMX detection (Table 1), the proposed MIP/CdTe QDs-decorated POF MP sensor offers a low detection limit and pays equal attention to a wide linear detection range and a better stability simultaneously. Moreover, it is simpler, faster, more cost-effective and it only requires a small amount of the solution to be tested (covering the microprobe). Furthermore, the introduction of MIP in this sensor structure enhances the specificity of this method, while the use of CdTe QDs contribute to the improved sensitivity. Last but not least, the integration of MIP-QDs with the POF MP allows for pollution-free, residue-free and *in-situ* detection, making it more versatile and flexible for different application scenarios. This proposed MIP/CdTe QDs-decorated POF MP can serve as a simple, rapid, flexible, cost-effective, sensitive, and specific approach for accurately detecting AMX concentrations in solution.

**TABLE 1:** Comparison of the proposed MIP/CdTe QDs-decorated POF MP sensor with other methods for AMX detection.

Detection technique	Linear range (μg/L)	LOD (μg/L)	Ref.
Chromatography	1.50–10000.00	3.00	Riezk et al. (2023)
Electrochemistry	328.90–25212.60 913.50–20827.80	18.30 274.05	Wong et al. (2020) Lopez et al. (2021)
SPR	0.04–0.95	0.03	Ayankojoa et al. (2018)
Boron-doped Carbon QDs MIP& CdTe QDs MIP/CdTe QDs-decorated POF MP	522.52–156800.45 0.20–50.00 0.50–50.00	301.46 0.14 0.31	Zhang et al. (2020) Chullasat et al. (2018) This work

## 3 Conclusion

In this study, a novel MIP/CdTe QDs-decorated POF MP was successfully developed for the *in-situ* detection of AMX with excellent safety, sensitivity, reversibility, reusability, specificity, performance and structure stability. The combination of MIP and CdTe QDs enabled the creation of highly specific recognition sites for AMX, achieving a targeted identification. The solid integration of the MIP-QDs with the flexible and compact POF MP added more exceptional performance in *in-situ* detection, reusability, pollution-free and residue-free operation, structure stability and biosafety. Moreover, the experimental results indicated the proposed sensor had a high sensitivity and low LOD (0.31 μg/L) at a linear detection range of 0.5–50 μg/L. We believe that the proposed sensor structure and method will have a great potential in developing and promoting integrated biosensing devices for the application in drug discovery, pharmaceutical sensing, biomedical diagnostics, environmental monitoring and food safety.

## 4 Methods

### 4.1 Synthesis of MIP-coated CdTe QDs (MIP-QDs)

MIP-QDs were synthesized via a sol-gel copolymerization process. Firstly, 1 mg of AMX (template molecule) was dissolved in 250 μL of deionized water and then mixed with 5 μL of γ-Aminopropyl triethoxysilane (APTES, functional monomer) in a brown vial. The above mixed solution was stirred at room temperature (25 °C) for 1 h. Subsequently, 100 μL of mercaptopropionic acid (MPA)-capped CdTe QDs (10 mg/mL), 30 μL of Tetraethyl orthosilicate (TEOS, cross-linking agent), and 50 μL of 3% ammonia solution (activator) were added into the mixed solution, which was then followed by continuous stirring for 6 h. The resulting product was collected by centrifugation at 3500 RCF for 10 min and washed three times with ethanol to remove the AMX template molecules; thus, MIP-coated CdTe QDs with the specific recognition cavities of AMX were successfully synthesized. Finally, the obtained MIP-QDs were dried in an oven at 40 °C for 1 h and then ready for the later use. As a comparison, non-imprinted polymer-coated QDs (NIP-QDs) were also synthesized using the same procedure but without the addition of AMX template molecules.

## Data Availability

The original contributions presented in the study are included in the article/supplementary material, further inquiries can be directed to the corresponding author.
